# Effect of lactoferrin in oral nutrition supplement (ONS) towards IL-6 and IL-10 in failure to thrive children with infection

**DOI:** 10.12688/f1000research.130176.3

**Published:** 2023-12-19

**Authors:** Nur Aisiyah Widjaja, Azizah Hamidah, Marissa Tulus Purnomo, Eva Ardianah

**Affiliations:** 1 Child Health, Airlangga University, Surabaya, East Java, 60286, Indonesia

**Keywords:** IL-6, IL-10, lactoferrin, growth failure

## Abstract

**Background:**

Growth failure due to infection in children is a major health problem throughout the world. It provokes a systemic immune response, with increased interleukin (IL)-6 and reduced IL-10. Lactoferrin (Lf) is a multifunctional iron-binding protein that can be found in whey protein inside formula milk such as oral nutrition supplement (ONS), which is able to upregulate anti-inflammatory cytokines (IL-10) and modulate pro-inflammatory cytokines. We conducted this study to investigate the effect of Lf supplementation in ONS on IL-6 and IL-10 levels in children with failure to thrive and infection.

**Methods:**

We performed a quasi-experimental pre- and post-study in children aged 12–60 months old with failure to thrive due to infectious illness. The subjects received 400 ml of oral nutritional supplements (ONS, 1 ml equivalent to 1 kcal) each day for 90 days, and their parents received dietary advice and medication based on the underlying illness. Blood was drawn to measure IL-6 and IL-10 before and after the intervention.

**Results:**

There were 75 subjects recruited and divided into group-1 and group-2 based on age. The incidence of undernutrition was 37.33%. Lf in ONS intervention improved body weight and body length. Lf also reduced IL-6, although there was not a significant difference before and after the intervention. However, the IL-6 reduction was significantly higher in subjects with undernutrition compared with subjects with weight faltering. Pre-intervention IL-6 levels were higher in children with stunting than in children with normal stature. There was a greater change in IL-6 in children with severe stunting than in children with normal stature or stunting. IL-10 was significantly reduced after the intervention.

**Conclusions:**

In addition to improving body weight and length, Lf supplementation in ONS improved immune response homeostasis by balancing IL-6 and IL-10 levels and by improving the IL-6/IL-10 ratio.

ClinicalTrials.gov number ID: NCT05289674, dated May 3 ^rd^ 2022.

## Introduction

Growth failure is an important health problem, with weight-for-age z-score (WAZ) and length-for-age z-score (LAZ) declining during the golden period or *golden 1,000 days* (period during pregnancy until second years of life), ^1^ and insignificant growth thereafter. ^2^ Nutritional intervention during this period will impact a child’s growth, development and ability to thrive. ^1^ Infection in children causes growth failure by provoking a systemic immune response which affects the nutritional status, ^3^ especially as a result of a reduction of insulin-like growth factor 1 (IGF-1). ^4^

Undernutrition refers to children who are underweight, stunted, or wasted, or those with nutrient deficiencies, which render them vulnerable to infections. ^5^ Stunting, defined as a height-for-age z-score (HAZ) or length-for-age z-score (LAZ) less than -2 standard deviations (SD), ^6^^,^^7^ is a linear growth failure resulting from chronic malnutrition that is irreversible. Wasting, characterized by being too thin, is defined as a weight-for-height z-score (WHZ) less than -2 SD, representing acute malnutrition, while underweight is identified by a low weight-for-age z-score (WAZ) using the WHO child growth standards as the indicator. ^8^ The prevalence of stunted and severely stunted children under two-years-old was 33.7% ^9^ and 45.4% in Nigerian children, ^10^ which was higher compared to this study. While the prevalence of underweight in two- to five-year-old children population in Gaza was 19.6%. ^11^ The prevalence of stunted/severely stunted children was higher in group-1 compared to group-2 in our study, which was similar to the study conducted in Nigeria, accounting for 45.5% vs. 12.2%. ^10^ However, a study in West Sulawesi, Indonesia found that children aged two to five years had a higher incidence of stunted/severely stunted growth compared to children aged one- to two-years-old, 33.64 vs. 23.12%. ^12^

Undernutrition leads to a chronic inflammation through immune defects, resulting in recurrent infections. ^13^ This immune alteration is known to be associated with an increased mortality risk in undernourished children. ^14^ The interaction between undernutrition and infections occurs in two ways: undernutrition heightens children's susceptibility to infection, and infection, in turn, contributes to undernutrition. ^15^ In particular, infection inhibits or slows growth velocity, leading to stunting. ^16^ Moreover, infection induces the acute phase response, suspected as a cause of stunting. ^17^ The induction of the acute phase response and the production of proinflammatory cytokines by infection directly impact bone remodeling, crucial for long bone growth, ^3^ and also inhibit chondrogenesis. ^18^ Proinflammatory cytokines such as interleukin (IL)-1β, IL-6, and tumor necrosis factor-alpha (TNF-α) have been found to be elevated in stunted children, leading to increased leptin levels and a subsequent decrease in appetite. ^4^ These proinflammatory cytokines also contribute to bone breakdown. ^18^ IL-6, with both anti- and pro-inflammatory functions, stimulates hepatocytes in the liver to produce acute-phase proteins and cytokines via multiple signaling pathways when it binds with IL-6 receptors. ^19^

Interleukin-10 (IL-10) is an anti-inflammatory cytokine with essential roles. ^20^ It functions to dampen and minimize damage resulting from the pro-inflammatory response ^21^ by inhibiting the activation of lymphocyte, thereby terminating the immune reaction. ^22^ An animal study using malnutrition models indicated that IL-10 levels remain within the normal range in malnourished mice but are elevated in kwashiorkor mice, ^23^ which supports by another finding. ^24^ Malnutrition downregulates type 1 cytokines (IL-2, IFNγ) but upregulates type 2 cytokines (IL-4 and IL-10), as observed in marasmic children. ^22^ This condition will make undernourished children become more susceptible to infections, with the potential for fatality leading to death. The risk of infection correlates directly with malnutrition degree, in which children with WAZ or HAZ below -3 SD have a 37% risk of diarrhoea. ^25^

The prevalence of tuberculosis (TB) is high in Indonesia, with an increase observed during 2017-2019, rising from 429,219 to 523,614 individuals, or from 167 per 100,000 to 196 per 100,000. ^26^ The manifestation of TB in children varies, depending on the type of TB, such as the presence of chronic cough, fever, weight loss, or failure-to-thrive. ^27^ The burden of TB in the pediatric population remains significant, with 1.2 million out of 10 million children affected by TB, resulting in a mortality rate of 16%. ^28^ Urinary tract infection (UTI) contributes to the incidence of stunting in children, as it causes anorexia, leading to a stagnant or inadequate weight gain. ^29^

Oral nutritional supplements (ONS), also known as food for special medical purposes, contains both macro- and micronutrients that are sufficient to meet daily nutritional needs for those at risk of malnutrition. ^30^ ONS is prescribed to increase nutritional intake due to insufficiency in diets to meet daily nutritional requirements, ^31^ particularly protein and calories. ^32^ ONS not only offers benefits for hospitalized patients, including a reduced length of hospital stay (LOS), lower inpatient costs, decreased complication rates, and rates of readmission, ^33^ but it also enhances lean body mass recovery. Furthermore, ONS contributes to improved energy intake and positively influences nutritional indicators such as body weight, length, and mid-arm circumference. ^34^ For children, ONS is a dairy milk-based product, which is designed to provide an energy density of 1–1.5 kcal/ml, and is expected to be effective to improve growth. ^35^

Lactoferrin (Lf) concentration within the whey protein that is contained in the modified cow’s milk formula is only 0.1 mg/ml. ^36^ ONS contains 10.8 g of protein per 100 g, comprising 46% whey and 54% casein. The effect of Lf supplementation (dose 0.6 g/L and 1.00) compared to standard infant formula on body weight showed no significant difference in children until 12 months old. Lf acts as an innate immune regulator and defense mechanism due to its antimicrobial properties. ^38^ As an immunodulatory, Lf acts by balancing the regulation of innate and adaptive immune cells (up- or down-regulate) to create the immune homeostasis. ^38^ Lf can also interact with the immune system, such as influencing cytokine activity by upregulating anti-inflammatory cytokines (IL-4 and IL-10) or modulating proinflammatory cytokines. ^39^ An *in vitro* study demonstrated that administering Lf at a dosage of 10 mg/mouse intravenously before thymectomy resulted in a 70% reduction in IL-6 and a 30% reduction in TNF-α levels four hours after the operation. ^40^ A study in adults showed that Lf reduced systemic inflammatory biomarkers by 61%, improved immune function by 75%, changed immune cell activity by 40% and reduced respiratory tract infection outcome by 60%. In adults, lactoferrin has been shown to reduce IL-6 by 24.9 pg/mL. ^41^

Here, we investigate the effect of lactoferrin in ONS towards IL-6 and IL-10 in failure to thrive children with infections during a 90-day intervention. Our hypothesis is that lactoferrin influences the immune systems of children with failure to thrive and infections, particularly in relation to IL-6 and IL-10.

## Methods

### Ethical statement

The study passed ethical exemption and was declared to be ethically appropriate by the Health Research Ethics Committee, Airlangga University, Surabaya, Indonesia, number 226/EC/KEPK/FKUA/2021 on October 4 ^th^ 2021 and registered on ClinicalTrials.gov number ID: NCT05289674, initial released on May 3 ^rd^, 2022.

### Participants

A quasi experimental pre- and post- study design was performed from October 2021 until July 2022 recruiting children aged between 1 years (12-months-old) and 5 years (60-months-old) with failure to thrive due to infectious illness (mainly urinary tract infection and tuberculosis (TB)) diagnosed by a paediatrician (the researcher) based on clinical and laboratory findings at Husada Utama Hospital outpatient unit, Surabaya, Indonesia. The subjects included in the study were excluded if they had fluid retention, organomegaly, a tumor mass, congenital abnormalities, cerebral palsy or hormonal disorders and syndromes. A written informed consent was signed by the parents as approval to participate in the study after the researcher explained the importance, the risks and the benefits of this study.

### Sample size

The sample size was determined using the formula below:

$$n1=n2{\left[\frac{\left(Z\propto \sqrt{2\mathrm{PQ}}+\mathrm{Z\beta}\;\sqrt{P1Q1+P2Q2}\right)}{\left(P1-P2\right)}\right]}^{2}$$

Note:

*N* = sample size

*Zα* = standard deviation (α) 5% (1.96)

*Zβ* = power, the researchers determined 90% (0.842)

*P*1 = clinical judgement 15% = 0.1

*P*2 = standard effect *P*2 = 25.5% = 0.25. ^42^

*P* = ½ ( *P*1+ *P*2) = 0.175

*Q* = 1- *P* = 0.825

*Q*1 = 1- *P*1 = 0.9

*Q*2 = 1- *P*2 = 0.75

The sample size was 80 subjects; pre- and post-design needed for the study was 80 samples.

### Interventions

The subjects were given an oral nutritional supplement or ONS with Lf (1 ml ~1 kcal, 400 ml/day), SGM Eksplor Gain Optigrow ^®^ prescribed by the researchers for 15 days consumption (equal to 4 boxes of 400 g) for initial intervention to detect any adverse reaction. The authors used this formula due to its relatively cheaper cost compared to other high calorie formula (ONS) available in Indonesia. The parents also had dietary counseling, animal protein was provided and a medication plan was given according to the underlying disease. Parents were asked to report any side effects to the researcher’s team by phone for further medical treatment. The parents were asked to visit the doctor after 14 days of ONS consumption for anthropometric measurements, compliance and side effect monitoring at day 15. While visiting, parents also received ONS for the next 15 days’ consumption (day 16 to 30), and were asked to visit the doctor again on day 30, 60 and 90. At the 30-day visit, parents received ONS for two months’ consumption (day 31 to day 60, and day 61 until day 90) (8 boxes of 400 g) and anthropometric measurements.

Blood was withdrawn via vena cubiti by a laboratory employee at Husada Utama Hospital to measure IL-6 (human IL-6 ELISA kit, code E0090Hu, BT Lab) and IL-10 (human IL-10 ELISA kit, code E0102Hu, BT Lab) before (day 0, when the parents agreed to participate in this study) and after the intervention (day 90). After the blood samples were collected, they were placed in a non-EDTA containing tube for micro-centrifugation to separate blood plasma from blood serum at 3000 rpm for 10 minutes. The supernatant was removed and placed in a PCR tube of 1.5 mL, then kept in a freezer at -4°C. Due to the researchers working during evening until night, the subjects were taken the blood at that time by the laboratory employee accompanied by the doctor’s nurses without fasting.

An indirect sandwich ELISA was performed to analyse IL-6 and IL-10 levels before- and after nutritional intervention using blood serum. For the sandwich ELISA, all reagents (standard solution, wash buffer, substrate solution A, substrate solution B and stop solution) were brought to room temperature before use (27°C). ^43^

#### Preparation of standard solution

A total of 120 μL standard solution (640 ng/ml) was diluted with 12 μL standard diluent to produce a 320 ng/L standard stock solution, and it was then allowed to rest for 15 minutes. Standard duplication points were made using a serial dilution of standard stock solution to produce 160 ng/L, 80 ng/L, 40 ng/L and 20 ng/L solutions.

#### Preparation for wash buffer solution

Then 20 ml of wash buffer concentrate 25 × was added to distilled water to yield 500 mL of 1 × wash buffer. The wash buffer was mixed gently if crystals formed in the concentrate until the crystals had completely dissolved.

#### Assay procedure

The assay procedure was performed at room temperature after we determined the number of strips required for the assay, and then we inserted the strips in the frames for use.

1. 50 μL of the standard solution was added into all the sample wells.
2. Then 50 μL standard solution was added into the standard wells.
3. 40 μL of sample was added to the sample wells and then 10 μL of human IL-6 or IL-10 antibody was added. Then 50 μL streptavidin-HRP was added to sample wells and standard wells, but not the blank control well. Each of them was mixed before the wells were placed on the plate and then sealed for incubation at 37°C for 60 minutes.
4. After 60 minutes of incubation, the seals were removed, and the plates were washed 5 times with wash buffer; the wells were soaked in 300 μL of wash buffer for 30 seconds to 1 minute for each wash.
5. 50 μL of substrate solution A and 50 μL of substrate solution B were added to each well and the plate was covered and incubated for 10 minutes at 37°C in the dark.
6. 50 μL of stop solution was added to each well, so that the blue colour changed to yellow immediately.

We then determined the optical density (OD value) of each well immediately using a microplate reader set at 450 nm of wavelength within 30 minutes after the stop solution was added, and then the standard curve were made. ^43^

Body weight was measured using a Seca 354 digital baby scale or a Seca 813 electronic flat scale) and body length/height was measured using a Seca 415 infantometer or Seca 213 stadiometer). Both measurements were taken twice by a trained nurse in the outpatient department of Husada Utama Hospital. The weight and length/height were the average value of the two measurements. When the subjects were weighed and measured, they wore light clothes without footwear or hair accessories. Anthropometry measurement for weight-for-age z-score (WAZ), length-for-age or height-for-age z-score or height-for-age z-score (LAZ/HAZ) and weight-for-length or weight-for-height z-score or weight-for-height z-score (WLZ/WHZ) were determined using WHO Anthro offline version 3.2.2. All the data was summarized in underlying data ^44^ and extended data. ^45^

### Statistical analysis

Statistical analysis conducted in this study was a test of normality and homogeneity, independent sample T-test or Mann-Whitney U test, Fischer exact test, Pearson chi-square, paired sample T-test or Wilcoxon, two-way ANOVA and one-way ANOVA using IBM SPSS Statistics version 21.

We hypotheses that:

• Lactoferrin in ONS improves body weight and length/height.
• Lactoferrin supplementation in ONS improves immune response homeostasis by balancing IL-6 and IL-10 levels, and by improving the IL-6/IL-10 ratio.
• At least there was a pair of groups of nutritional status had a significant difference of IL-6 and IL-10 levels, and the IL-6/IL-10 ratio.

## Results

**Figure 1.**
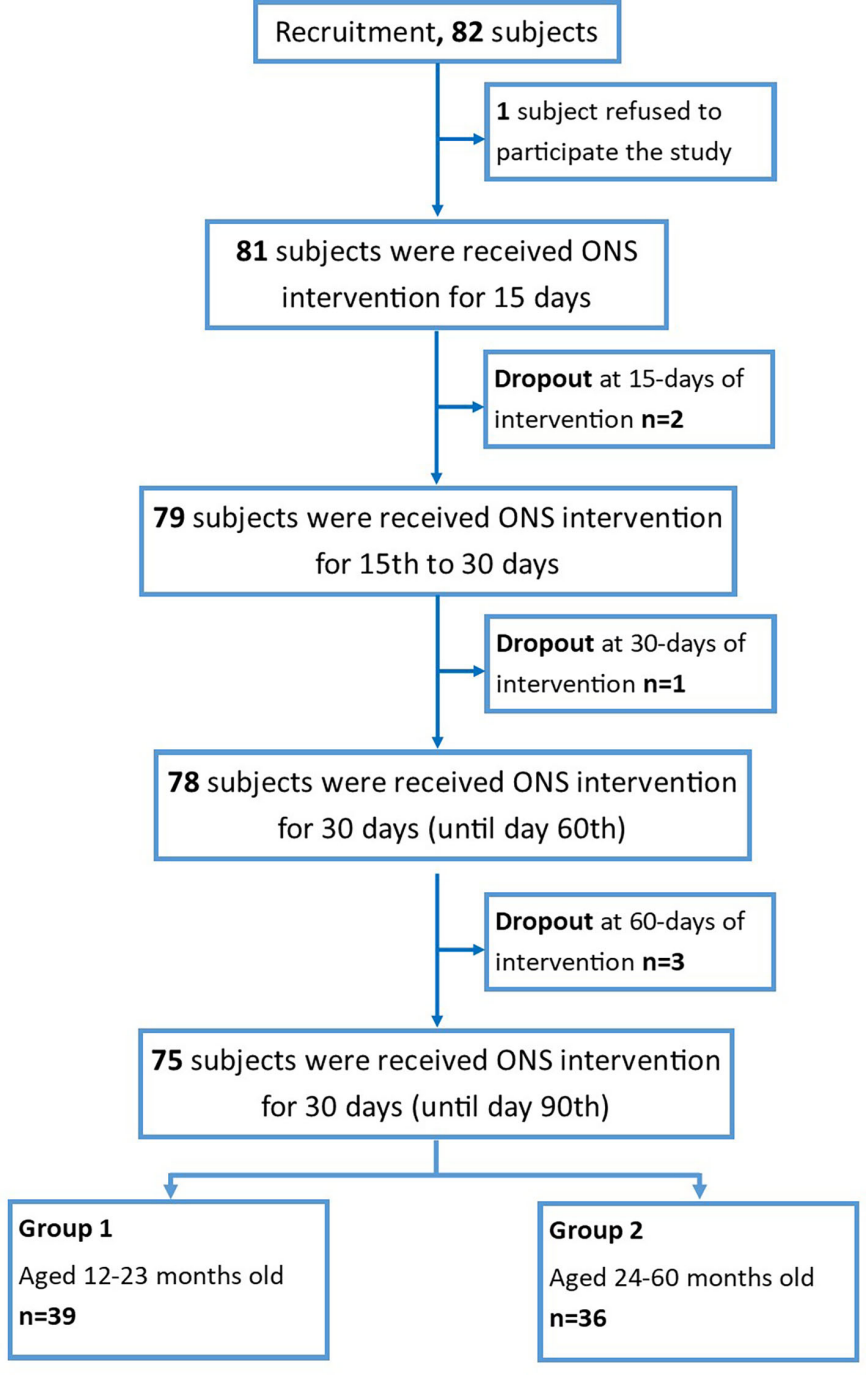
Flowchart of the subject’s recruitment.

Seventy-five subjects were involved in the study and divided into two groups based on the age of the participant: group-1 (age 1–2 years, n=39) and group-2 (age 2–5 years old, n=36), as summarized in Figure 1.

Table 1 summarizes the characteristics of the subjects who participated in the study. The ratio of male/female was 12/13 and there was no significant difference in gender distribution in both groups (p = 0.108). There was no significant difference in the main complaint (p = 0.229), duration of complaints (p = 0.580), WAZ (p = 0.482) and WLZ/WHZ (p = 0.499). Age, ideal body weight and height age were lower in group-1 compared to group-2 (p <0.05). LAZ was lower in group-1 compared to group-2 (-1.95 ± 1.17 vs. -1.19 ± 0.86, p = 0.002).

**Table 1.** Subject’s characteristic during the study.

Subject’s characteristics	Group-1 (mean ± SD)	Group-2 (mean ± SD)	p
Age, months	16.74 ± 3.63	35.44 ± 7.70	0.000 ^1^
Gender, n (%)			0.108 ^2^
- Male	15 (36.59)	21 (58.33)	
- Female	24 (58.53)	15 (41.67)	
Main complaints:			0.229 ^3^
- Feeding difficulties with stagnant body weight gain	10 (25.64)	16 (44.44)	
- Stagnant body weight gain and length gain	1 (2.56)	0	
- Body weight gain	27 (69.29)	20 (55.56)	
- Fever with watery stools	1 (2.56)	0	
Duration of complaint, months	7.84 ± 4.29	12.23 ± 11.15	0.580 ^1^
WAZ	-1.52 ± 1.08	-1.35 ± 0.99	0.482 ^4^
WAZ categories, n (%)			0.874 ^3^
- Weight faltering (normo-weight)	25 (64.10)	21 (58.33)	
- Underweight	12 (30.77)	13 (36.11)	
- Severely underweight	2 (5.13)	2 (5.56)	
LAZ/HAZ	-1.95 ± 1.17	-1.19 ± 0.86	0.002 ^4^
LAZ/HAZ categories, n (%)			0.004 ^3^
- Normo-stature	17 (43.59)	29 (80.56)	
- Stunted	14 (35.90)	5 (13.89)	
- Severely stunted	8 (20.51)	2 (5.56)	
WLZ/WHZ	-0.85 ± 0.92	-1.01 ± 1.12	0.499 ^4^
WLZ/WHZ categories, n (%)			0.486 ^3^
- Good nutrition	35 (89.74)	29 (80.56)	
- Wasted	3 (7.69)	6 (16.67)	
- Severely wasted	1 (2.56)	1 (2.78)	

Note: WAZ = weight-for-age z-score; LAZ = length-for-age z-score; HAZ = height-for-age z-score; WLZ = weight-for-length z-score; WHZ = weight-for-height z-score.

^1^ Mann-Whitney U Test.

^2^ Fischer’s Exact Test.

^3^ Pearson Chi Square.

^4^ Independent Sample T-Test.

Table 2 summarized the test of normality and homogeneity for each variable which was needed for further statistical test. It showed that body weight, body length/height, WAZ, LAZ/HAZ, and WLZ/WHZ had normal distribution and homogenous, so the independent sample T-test and oneway anova could be ruled out.

**Table 2.** Test of normality and homogeneity.

Variables	Kolmogorov-Smirnov p value	Interpretation	Levene’s test of homogeneity	Interpretation
Body weight before the intervention	0.200	Normal distribution	0.077	Homogenous
Body weight after the intervention	0.187	Normal distribution	0.148	Homogenous
Body length/height before the intervention	0.200	Normal distribution	0.659	Homogenous
Body length/height after the intervention	0.200	Normal distribution	0.853	Homogenous
WAZ	0.200	Normal distribution	0.726	Homogenous
LAZ/HAZ	0.200	Normal distribution	0.158	Homogenous
WLZ/WHZ	0.200	Normal distribution	0.285	Homogenous
IL-6 before	0.001	Abnormal distribution	0.851	Homogenous
IL-6 after	0.017	Abnormal distribution	0.619	Homogenous
ΔIL-6	0.002	Abnormal distribution	0.733	Homogenous
IL-10 before	0.003	Abnormal distribution	0.682	Homogenous
IL-10 after	0.051	Normal distribution	0.650	Homogenous
ΔIL-10	0.007	Abnormal distribution	0.392	Homogenous
IL6/IL-10 ratio before	0.000	Abnormal distribution	0.185	Homogenous
IL6/IL-10 ratio after	0.006	Abnormal distribution	0.794	Homogenous
ΔIL6/IL-10 ratio	0.000	Abnormal distribution	0.538	Homogenous

The incidence of underweight and severely underweight children in group-1 and group-2 was 33.33% and 5.33% respectively, and there was no significant difference in WAZ categories in both groups (p = 0.874). The incidence of stunted and severely stunted children in group-1 and group-2 was 25.33% and 13.33%, respectively, demonstrating a higher prevalence of stunted/severely stunted children in group-1 compared to group-2 (56.41% vs. 19.45%, p = 0.004). However, the incidence of stunted/severely stunted children in group-1 was predominantly boys (6 boys vs. 1 girl). The incidence of wasted and severely wasted children in group-1 and group-2 were 12% and 2.67% (p = 0.486).

The effect of ONS on body weight and body length/height change is summarized in Table 3. Initial body weight before treatment was lower in group-1 compared to group-2 (p = 0.000). post intervention body weight was lower in group-1 than in those of group-2 (p = 0.000) but the weight change (Δ body weight) in both groups showed no significant difference (922.56 ± 671.28 vs. 855.55 ± 577.16 g, p >0.05). The initial body length/height was shorter in group-1 compared to group-2 (p = 0.000), so the late body length/height was shorter in group-1 compared to group-2 (p = 0.000). Body length/height change was greater in group-1 compared to group-2 (3.49 ± 1.43 vs. 2.08 ± 1.04 cm, p = 0.000).

**Table 3.** Body weight (in g) and body length/height (in cm) changes after intervention.

Parameters	Group-1 (mean ± SD)	Group-2 (mean ± SD)	p
Initial body weight	8,735.38 ± 1,318.64	12,164.17 ± 1,688.89	0.000 ^1^
Late body weight	9,657.95 ± 1,607.48	13,019.72 ± 1,768.91	0.000 ^1^
Body weight change (Δ Body weight)	922.56 ± 671.28	855.55 ± 577.16	0.646 ^1^
Initial body length/height	75.14 ± 5.26	91.39 ± 4.80	0.000 ^1^
Late body length/height	78.63 ± 5.02	93.47 ± 4.84	0.000 ^1^
Body length/height change (Δ length/height)	3.49 ± 1.43	2.08 ± 1.04	0.000 ^1^

^1^ Independent sample T-test.

IL-6 and IL-10 levels during the intervention are summarized in Table 4. The levels of IL-6 post-intervention (day 90) were not significantly different from pre-intervention (128.45 ± 109.92 vs. 111.76 ± 78.10 pg/mL, p = 0.554), although there was a decline (-16.68 ± 91.09 pg/mL) in both groups (-13.42 ± 97.80 vs. -20.23 ± 84.46 pg/mL, p = 0.749). There was no significant difference in IL-6 levels before the treatment in both groups (p <0.232) and after treatment (p <0.191). IL-10 level was significantly reduced after the intervention (461.20 ± 392.12 became 261.28 ± 163.97 pg/mL, Δ = 199.92 ± 339.01 pg/mL, p = 0.000). The reduction in IL-10 showed no significant difference in either group (-183.24 ± 378.50 vs. -217.99 ± 294.61 pg/mL, p = 0.518).

**Table 4.** IL-6 and IL-10 in both group-1 and group-2 (in pg/mL).

IL-6 markers	Group-1 (mean ± SD)	Group-2 (mean ± SD)	p
Initial levels of IL-6	113.80 ± 109.24	144.31 ± 109.96	0.232 ^1^
Initial levels of IL-6 based on nutritional status			0.669 ^2^
- Weight faltering	94.12 ± 85.49	126.13 ± 93.17	
- Undernutrition	136.76 ± 130.54	191.58 ± 139.54	
Late levels of IL-6	100.38 ± 73.95	124.09 ± 81.59	0.191 ^**1**^
Late levels of IL-6 based on nutritional status			0.121 ^2^
- Weight faltering	95.31 ± 70.06	115.52 ± 76.85	
- Undernutrition	108.51 ± 81.65	166.89 ± 98.51	
Δ IL-6	-13.42 ± 97.80	-20.23 ± 84.46	0.749 ^**1**^
Δ IL-6 levels based on nutritional status			0.001 ^2^
- Weight faltering	-9.00 ± 83.72	-19.86 ± 64.76	
- Undernutrition	-18.56 ± 114.38	-21.18 ± 126.85	
Initial levels of IL-10	423.66 ± 385.66	501.88 ± 400.43	0.392 ^**1**^
Initial levels of IL-10 based on nutritional status			0.131 ^2^
- Weight faltering	407.09 ± 367.31	411.74 ± 318.22	
- Undernutrition	442.99 ± 415.93	736.23 ± 507.72	
Late levels of IL-10	240.41 ± 165.75	283.89 ± 161.27	0.254 ^**1**^
Late levels of IL-10 based on nutritional status			0.650 ^2^
- Weight faltering	211.15 ± 100.40	256.25 ± 137.08	
- Undernutrition	274.56 ± 217.39	355.76 ± 202.48	
Δ IL-10	-183.24 ± 378.50	-217.99 ± 294.61	0.661 ^**1**^
Δ IL-10 levels based on nutritional status			0.113 ^2^
- Weight faltering	-195.94 ± 345.44	-155.49 ± 234.96	
- Undernutrition)	-168.43 ± 423.58	-380.47 ± 378.91	
Initial value of IL-6/IL-10 ratio	0.33 ± 0.28	0.33 ± 0.14	0.991 ^1^
Initial value of IL-6/IL-10 ratio based on nutritional status			0.179 ^2^
- Weight faltering	0.28 ± 0.17	0.34 ± 0.16	
- Undernutrition	0.38 ± 0.37	0.29 ± 0.16	
Late value of IL-6/IL-10 ratio	0.43 ± 0.12	0.45 ± 0.12	0.616 ^1^
Late value of IL-6/IL-10 ratio based on nutritional status			0.560 ^2^
- Weight faltering	0.43 ± 0.12	0.44 ± 0.11	
- Undernutrition	0.44 ± 0.11	0.47 ± 0.12	
Δ IL-6/IL-10 ratio	0.12 ± 0.29	0.12 ± 0.17	0.262 ^1^
Δ IL-6/IL-10 ratio based on nutritional status			0.125 ^2^
- Weight faltering	0.15 ± 0.19	1.00 ± 0.16	
- Undernutrition	0.06 ± 0.37	0.18 ± 0.21	

^1^ Independent sample T-test.

^2^ Two-way ANOVA.

There was significantly improvement in the IL-6/IL-10 ratio after the intervention (0.33 ± 0.22 vs. 0.44 ± 0.12, Δ = 0.11 ± 0.23, p = 0.000). The reduction in the IL-6/IL-10 ratio showed no significant difference in either group (0.11 ± 0.29 vs. 0.12 ± 0.17, p = 0.991).

The levels of IL-6 based on anthropometric categories are summarized in Table 5. Based on the LAZ categories, there was a significant difference in IL-6 levels pre-intervention (p = 0.045), in which the stunted group had higher levels of IL-6 compared to those with a normal stature (212.06 ± 146.05 vs. 115.81 ± 93.84 pg/mL, p = 0.037). Although there was no significant difference, IL-6 was higher in the stunted group compared to those who were severely stunted (212.06 ± 146.05 vs. 85.45 ± 89.06 cm, p = 0.057). There was no significant difference in post-intervention levels of IL-6 (p = 0.083); however, IL-6 was lower in normal stature children compared to stunted and severely stunted children. Although IL-6 levels were higher in the stunted group compared to the severely stunted group, there was no significant difference between both groups (212.06 ± 146.05 vs. 85.45 ± 89.06 pg/mL, p = 0.057). Changes in IL-6 (ΔIL-6) based on the LAZ/HAZ categories showed no significant difference (p = 0.055), but the changes in severely stunted children were higher compared to the stunted group (47.33 ± 93.48 vs. -41.66 ± 108.69 pg/mL, p = 0.036) and the normal stature group (47.33 ± 93.48 vs. -20.28 ± 77.36 pg/mL, p = 0.031). This was due to an increase in IL-6 levels in severely stunted children, while stunted children and those of normal stature exhibited reduced IL-6 levels.

**Table 5.** IL-6 levels based on anthropometry categories.

Markers	Pre-intervention (mean ± SD)	Post-intervention (mean ± SD)	ΔIL-6 (mean ± SD)
IL-6 based on LAZ/HAZ categories			
- Normo-stature	115.81 ± 93.84	104.81 ± 48.67	-20.28 ± 77.36
- Stunted	212.06 ± 146.05	117.51 ± 115.94	-41.66 ± 108.69
- Severely stunted	85.45 ± 89.06	132.78 ± 103.03	47.33 ± 93.48
p value	0.045 ^2^	0.083 ^1^	0.055 ^2^
IL-6 based on WAZ categories			
- Normo-weight	125.59 ± 99.41	109.49 ± 75.87	-16.09 ± 73.95
- Underweight	145.73 ± 151.51	101.54 ± 73.55	-44.19 ± 137.03
- severely underweight	103.70 ± 85.38	181.84 ± 111.85	78.14 ± 32.06
p value	0.903 ^2^	0.173 ^1^	0.014 ^2^
IL-6 based on WLZ/WHZ categories			
- Good nutritional status	131.54 ± 110.91	109.49 ± 75.98	-22.05 ± 92.83
- Wasted	129.02 ± 110.60	138.75 ± 97.46	9.73 ± 84.73
- Severely wasted	26.83 ± 5.26	63.04 ± 8.72	36.22 ± 3.46
p value	0.235 ^2^	0.391 ^1^	0.148 ^2^

Note: WAZ = weight-for-age z-score; LAZ = length-for-age z-score; HAZ = height-for-age z-score; WLZ = weight-for-length z-score; WHZ = weight-for-height z-score.

^1^ One-way ANOVA.

^2^ Kruskal-Wallis.

The levels of IL-6 based on the WAZ categories showed no significant difference pre-intervention (p = 0.903) or post-intervention (p = 0.173), but the change in IL-6 (ΔIL-6) showed a significant difference (p = 0.014), where the WAZ in severely underweight children increased, while the underweight and weight faltering decreased. Therefore, severely underweight children had higher changes of IL-6 compared to stunted children (78.14 ± 32.06 vs. -44.19 ± 137.03 pg/mL, p = 0.012) and weight faltering/normal weight children (78.14 ± 32.06 vs. -16.09 ± 73.95 pg/mL, p = 0.001).

The initial and late changes of IL-6 (ΔIL-6) based on WLZ/WHZ categories showed no significant difference (p >0.05). The initial level of IL-6 was higher in good nutritional status subjects compared to wasted and severely wasted. But after the intervention, IL-6 levels in good nutritional status subjects were reduced (-22.05 ± 92.83 pg/ml), while the wasted and severely wasted group increased (9.73 ± 84.73 and 36.22 ± 3.46 pg/ml respectively).

Initial and late levels of IL-10, and changes of IL-10 based on the anthropometric categories are summarized in Table 6. There was no significant difference in IL-10 before and after the intervention, or in changes of IL-10 (p <0.05) based on the LAZ/HAZ categories. A similar phenomenon was also seen in the WAZ and WLZ/WHZ categories (p <0.05).

**Table 6.** IL-10 levels based on anthropometry categories.

Markers	Pre-intervention	Post-intervention	ΔIL-6/IL-10 ratio
IL-10 based on LAZ/HAZ categories			
- Normo-stature (n = 52)	420.51 ± 350.98	242.98 ± 133.22	-177.53 ± 292.88
- Stunted (n = 13)	666.72 ± 451.21	302.92 ± 213.36	-363.80 ± 413.83
- Severely stunted (n = 10)	405.61 ± 469.84	302.30 ± 213.36	-103.31 ± 422.42
p value	0.114 ^1^	0.535 ^1^	0.076 ^2^
IL-10 based on WAZ categories			
- Weight faltering (normo-weight (n = 56)	437.99 ± 351.68	253.73 ± 148.63	-184.25 ± 297.31
- Underweight (n = 15)	497.15 ± 477.20	260.51 ± 216.90	-236.64 ± 440.80
- severely underweight (n = 4)	651.36 ± 630.80	369.88 ± 147.97	-281.48 ± 536.04
p value	0.538 ^1^	0.397 ^1^	0.956 ^2^
IL-10 based on WLZ/WHZ categories			
- Good nutritional status (n = 64)	457.82 ± 370.84	257.03 ± 159.36	-200.79 ± 331.63
- Wasted (n = 9)	571.20 ± 536.68	320.00 ± 201.23	-251.19 ± 421.01
- Severely wasted (n = 2)	74.55 ± 40.29	133.16 ± 25.89	58.61 ± 14.41
p value	0.268 ^1^	0.302 ^1^	0.156 ^2^

Note: WAZ = weight-for-age z-score; LAZ = length-for-age z-score; HAZ = height-for-age z-score; WLZ = weight-for-length z-score; WHZ = weight-for-height z-score.

^1^ One-way ANOVA.

^2^ Kruskal-Wallis.

The IL-6/IL-10 ratio based on anthropometric measurements is summarized in Table 7. The IL-6/IL-10 ratio based on the LAZ/HAZ categories showed no significant difference pre- and post-intervention. However, ONS supplementation increased the IL-6/IL-10 ratio in all LAZ/HAZ categories. In the WAZ categories, severely underweight children had a lower IL-6/IL-10 ratio compared to underweight children, even though there was no significant difference. The IL-6/IL-10 ratio increased after ONS therapy in all WAZ categories. A higher increment was seen in the severely underweight, but there was no significant difference. The WLZ/WHZ categories also showed no significant difference in the initial and late changes of the IL-6/IL-10 ratio.

**Table 7.** IL-6/IL-10 ratio based on anthropometric

Markers	Pre-intervention	Post-intervention	ΔIL-10
IL-6/IL-10 based on LAZ/HAZ categories			
- Normo-stature (n = 52)	0.32 ± 0.16	0.43 ± 0.11	0.12 ± 0.02
- Stunted (n = 13)	0.39 ± 0.38	0.45 ± 0.13	0.07 ± 0.41
- Severely stunted (n = 10)	0.30 ± 0.27	0.46 ± 0.11	0.15 ± 0.27
p value	0.390 ^2^	0.764 ^1^	0.698 ^1^
IL-6/IL-10 based on WAZ categories			
- Weight faltering (normo-weight (n = 56)	0.34 ± 0.24	0.44 ± 0.16	0.10 ± 0.26
- Underweight (n = 15)	0.30 ± 0.16	0.43 ± 0.11	0.12 ± 0.13
- severely underweight (n = 4)	0.19 ± 0.04	0.47 ± 0.16	0.28 ± 0.18
p value	0.141 ^2^	0.809 ^1^	0.340 ^1^
IL-6/IL-10 based on WLZ/WHZ categories			
- Good nutritional status (n = 64)	0.33 ± 0.23	0.44 ± 0.11	0.11 ± 0.25
- Wasted (n = 9)	0.28 ± 0.14	0.45 ± 0.12	0.17 ± 0.18
- Severely wasted (n = 2)	0.44 ± 0.31	0.49 ± 0.16	0.05 ± 0.15
p value	0.675 ^2^	0.804 ^1^	0.739 ^1^

Note: WAZ = weight-for-age z-score; LAZ = length-for-age z-score; HAZ = height-for-age z-score; WLZ = weight-for-length z-score; WHZ = weight-for-height z-score.

^1^ One-way ANOVA.

^2^ Kruskal-Wallis.

## Discussion

Stunted growth was found to be associated with age, and it was more prevalent in children aged less than 24-months-old. ^46^ Due to the incidence of stunted growth, which was higher in group-1, the LAZ value was significantly lower in group-1 compared to group-2. It was also found that children with stunted growth were significantly shorter in length/height than the control group in another study. ^47^ It was reported that children aged 12–23 months old had an increased risk of stunting by 1.8 times. ^9^^,^^48^

In group-2, the incidence of stunted/severely stunted growth was predominant in males, aligning with the findings of Akombi *et al*. (2017), which identified male sex as one of the risk factors for stunting in children aged 0–5 years. ^10^ This consistency with our study suggests that males may be more vulnerable to health inequalities. ^49^ The biological reason is due to the sex difference in the immune and endocrine systems, and testosterone, luteinizing hormone and follicle stimulating hormone are suspected to play a role. ^50^ Feeding practice preferences between boys and girls such as early weaning in boys, and a tendency for boys to consume more than one complementary feed meal within a 24-hour period, may also contribute to the observed patterns. ^51^

ONS intervention in undernourished or at nutritional risk children aged nine months to 12-years-old improved body weight by 0.423 kg after six months of intervention and height gain was 0.417 cm compared to the control, with greater gains in weight in the first 7–10 days of intervention (0.089 kg). ^52^ ONS improved growth in underweight children aged five- to 12-years-old after six and 12 months, ^53^ which was in line with this study, where both group-1 and group-2 gained weight. Formula feeding supplemented with lactoferrin is safe for infants under one year old with no difference in growth rate (g/day). ^37^

Lactoferrin intervention in children with diarrhoea aged 12–36 months old increased the LAZ/HAZ score (p = 0.03) compared to the placebo, ^54^ and the children also showed an increment in length/height. A similar result was also found in Vietnamese children aged 24–48 months old in a 12-month intervention. The intervention of 450 kcal of additional ONS during the first three months resulted in an increase in height of 1.62 cm, ^55^ which was lower than our results in a similar group (group-2). A higher calorie density intervention (2.4 kcal/ml vs. 1.5 kcal/ml) for 28 days increased the children’s height by 0.87 [0.59–1.16] and 0.55 [0.17–0.93] cm, p = 0.007 in children aged greater than one year and less than 12 years old with growth faltering. ^35^

Lactoferrin is known to have a bacteriostatic or bactericidal effect and it can activate the immune response of an organism, ^56^ and limits tissue damage due to excessive pro-inflammatory response (chronic response) caused by the infection. ^38^ It can therefore reduce the incidence of acute gastrointestinal symptoms and reduce the duration of respiratory symptoms in children under 12 months old due to viral or bacterial infection. ^57^ Regarding the immunological profile, when comparing an infant who received Lf supplementation vs. non-Lf supplementation vs. standard formula, although there was no significant difference between groups, there was an increase in TGF-β1 (6.5 vs. 4.3 vs. 2.8 ng/mL), TGF-β2 (0.26 vs. 0.26 vs. 0.22 ng/mL) and IL-2 (0.21 vs. 0.5 vs. 0.4 pg/mL), but a decrease in TNF-α (-2.4 vs. -1.5 vs. -1.7 pg/mL) during a four-month intervention. ^58^ A study that examined piglets with a 2 ml/day supplementation showed a decrease in bacterial colonies compared to those without Lf supplementation (1.109 x 10 ^7^ vs. 3.6183 x 10 ^8^ CFU) via an anal swab after a seven-day intervention. ^59^ It was stated that Lf induced the development of T cell helper type 1 (Th1) immunity, thus created the balance of monocytic pro- and anti-inflammatory cytokines. In a dose-dependent manner, Lf enhanced pro-inflammatory response *in vitro* (splenocyte and adherent (F4/80 ^+^) splenocyte populations, bone marrow derived monocytes (BMM), and J774A.1 cultured cells) and induced IL-12 and IL-10 production and increased the ratio IL-12:IL-10 in lipopolysaccharide (LPS) stimulated cells. ^60^ Studies of *Mycobacterium tuberculosis* infection treated with Bacillus Calmette–Guérin (BCG) and Lf emulsified with Freund’s adjuvant in mice showed a decreased mycobacterial load in the lungs and spleen. It also increased the protection against *M. tuberculosis*, ^37^^,^^61^^,^^62^ via downregulation of proinflammatory mediators (TNF-α, IL-1β) by modulation of macrophages and dendritic cell ability to present antigens and stimulate T-cells. Lf also increased IFNγ, which was the specific response towards Th1. ^38^ A study examining mice with urinary tract infection due to *Escherichia coli* showed that Lf intervention orally was able to decrease the number of bacteria in the kidneys and bladder after 24 h of Lf consumption, and reduced IL-6 by urinary leucocytes. ^63^ A study conducted on Senegalese children receiving tetanus vaccine in stunted children aged one- to nine-years-old showed that the production of IFNγ was compromised. ^2^ It was stated that undernutrition is related to immunodeficiency, even in mild cases, affecting both the innate and adaptive immune systems. ^2^

In our study, even though there was no significant difference in IL-6 levels before and after the intervention, Lf reduced IL-6, which is in line with other studies showing a reduction in undernutrition groups. It was found that IL-6 levels were lower in undernutrition compared to good nutrition groups (2.54 pg/mL vs. 6.02 pg/mL, p <0.0001). ^64^ Genetic investigation showed that the IL-6 164 gene with a GG and GC genotype (mutant phenotype) was more frequent in undernourished children. ^65^

When the groups were examined based on the LAZ/HAZ categories, stunted subjects had higher IL-6 levels compared to normal stature and there was a significant difference compared to severely stunted. This is in-line with a study in Egyptian children, where IL-6 was higher in stunted compared to normal stature children (1.6 ± 0.2 vs. 1.5 ± 0.3 pg/mL), ^66^ but it was decreased in malnourished compared to normal children. ^64^ This showed that when the children had an LAZ/HAZ score greater than or equal to -2 SD, IL-6 was increased but it decreased when children had an LAZ/HAZ score greater or equal to -3 SD. On malnutrition, the acute-phase response was attenuated, and the production of cytokines decreased. An animal study showed that IL-1β production decreased in malnourished guinea pigs induced with endotoxins. ^19^ Stunting is a form of growth failure due to long term nutritional deficiency or it is caused by chronic malnutrition or recurrent undernutrition. ^8^^,^^67^ After a six month intervention with food supplementation, stunted Bangladeshi children aged 12–18 months old experienced an IL-6 increment (from 0 [0–1.2] to 1.68 [0.83–4.7] pg/mL, p = 0.001), ^68^ which contradicts this study as IL-6 levels were reduced in stunted and normal stature children. However, IL-6 was found to have increased in severely stunted children, so the post-intervention levels of IL-6 were higher in severely stunted even-though there was no significant difference. Severely stunted children might undergo these immune alterations which are similar to severely acute malnutrition, so IL-6 levels were lowest at the outset but increased drastically after the intervention to surpass normal stature and stunted children. It was stated that immune function is an activity with high costs on energy demand, and in developing children the allocation of energy in immune functions may lead to a trade-off with physical growth, particularly those with exposure to infection. ^69^

A similar anomaly was also seen in the WAZ categories even though there was no significant difference. Being underweight has been used as an indicator of undernutrition due to a short term nutritional deficiency. ^8^ However, an *in vitro* study using peripheral blood mononuclear cells (PBMC) taken from children suffering from protein energy malnutrition (PEM) contradicted this study, which showed an increment in IL-6 expression after stimulation with LPS, ^70^ even though it was expressed earlier, reached its peak earlier, and lasted longer than controls in rats. ^71^ As the immune function is costly in terms of energy, it has negative effects on growth. In children with mildly elevated immune activity, they experience a growth reduction of up to 49%, ^69^ as seen in underweight children who experienced an increase in IL-6 due to the trade-off in body fat between immune function and growth. ^69^

Regarding malnutrition, lymphatic tissue, particularly the thymus, experiences atrophy, leads to a reduction in delayed-type hypersensitivity responses, followed by a reduction in levels of antibodies in severely malnourished children (≥-3 SD of WLZ/WHZ WHO child growth standards), but it remains intact in moderate malnutrition (leucocyte and lymphocyte, high levels of immunoglobulin, particularly IgA, and acute phase response), and cytokine patterns are skewed towards a Th2-response. ^14^ However, our study found that IL-6 started to reduce in wasted patients, with the lowest levels in those that were severely wasted. Nutritional intervention increases IL-6 in both wasted and severely wasted, but it is reduced with good nutrition, which is in line with research that states undernutrition, even in the mildest form causes immunodeficiency. ^13^

Wenling C57BL/6 J mice in a wasting models study which underwent 14 days of weight loss showed increases of IL-10 in the malnourished group at three and at 14 days. ^23^ It was stated that malnutrition modifies the body’s resistance against infection, particularly the immune response. Lipopolysaccharide (LPS) injection (1.25 μg i.v.) in a protein-energy malnutrition (PEM) mouse model, showed that the circulating levels of IL-10 were increased and high levels were found in bone marrow cells, which showed immunodeficiency. ^24^ This finding was in-line with a study in children with marasmic-PEM, IL-10 was significantly higher compared to controls (19.08 ± 5.93 vs 10.46 ± 3.90 pg/mL; p = 0.000). ^22^ This may be caused by the deficits of NF-kB activation. NF-kB was the major transcription pathway for proinflammatory cytokine production. ^72^ Using BMI as the parameter to determine malnutrition, subjects with severe malnutrition (BMI <16.5) had higher levels of IL-10 (8.0 ± 3.6 pg/mL) compared to those with moderate malnutrition (BMI = 16.5-18.4) (2.6 ± 4.3 pg/mL) and good nutrition (BMI ≥18.5) (2.8 ± 0.7 pg/mL) in adults, ^73^ which was similar to the WAZ category where IL-10 was slightly increased in those underweight, and increased drastically in those severely underweight.

Nutritional intervention increases IL-10 significantly in children aged 12–60 months old with moderate and severe malnutrition receiving curd (milk product) compared to leaf protein concentrate (LPC) (from 30.9 ± 29.5 to 67.4 ± 96.2 pg/mL vs. 29.2 ± 25.8 to 31.5 ± 24.9 pg/mL). Based on Gomez criteria for malnutrition severity, children with mild malnutrition had lower IL-10 compared to children with severe malnutrition. It was higher in subjects aged more than two years old compared to two- to five-year-olds due to a balancing pro-inflammatory response to minimalize tissue damage. ^74^ In malnourished children, IL-10 was found to be reduced, while in line with this study, IL-10 was depressed in severely wasted subjects. ^75^ However, the level of IL-10 was still normal in severely stunted or severely underweight children. The reduction is due to a deficiency in the number and functional Th cells, which may be caused by incomplete differentiation of T lymphocyte precursors and steroid-induced lympholysis. ^75^ Another study of malnourished children due to inadequate food intake (anorexia nervosa) and diarrhoea receiving nutritional intervention in the form of milk and yoghurt, showed increased IFNγ production post intervention, ^76^ which may be caused by incomplete differentiation of T lymphocyte precursors and steroid-induced lympholysis. In undernutrition subjects and weight faltering subjects, IL-10 tends to reduce, and a higher reduction was seen in undernutrition subjects, which showed that before intervention undernutrition subjects may experience immune alterations, as seen in the IL-6/IL-10 ratio, which was higher in undernutrition group-1, but lower in group-2. At post intervention, almost all the group had a similar value, ranging from 0.43 to 0.47. Adipose tissue is the main storage for nutrients, which can sense that nutrients are inadequate by releasing adipokines (particularly leptin) to control cellular metabolism and immune function. So, undernutrition has a direct impact on adipose tissue (volume and number), and directly influences the immune system. Leptin not only mediates glucose and lipid metabolism but also immune function, by stimulating activation, proliferation and production of pro-inflammatory cytokine (IL-6, TNF-α, monocytes, macrophages, dendritic cells, and NK cells). Leptin also promotes T-cell activation and development towards Th-1 and Th-17 cell subset which is proinflammatory. Regarding undernutrition, there was leptin depletion and in contrast adiponectin is produced, resulting in the polarization towards M2 or an alternative macrophage which then secretes IL-10 and IL-1Rα. This limits the activation of the NF-kB pathway, and reduces both T-cells or B-cells. Moreover, cortisol hormone restrains the generation of the proinflammatory immune response, so the ability of macrophages and neutrophils to infiltrate the infection site was also restrained. Proinflammatory cytokine production is also reduced, but anti-inflammatory cytokines (IL-10 and IL-33) are increased. ^5^

IL-6 has been used as a potential biomarker to identify patients receiving anti-inflammatory therapies as it is secreted widely as a response to pathological states such as infection, inflammation and cancer. IL-10 acts as an anti-inflammatory response, it is secreted as a response to dampen pro-inflammatory bursts and minimize tissue damage. The balance of IL-6 and IL-10 is an important biomarker reflecting the homeostasis of the immune response. In Covid-19 patients, each point increment of the IL-6/IL-10 ratio was associated with a 5.6 times more severe outcome. ^5^ In children with pneumonia, the IL-6/IL-10 ratio at 9.61 determines those with severe pneumonia to those with mild disease (sensitivity 76.5% and specificity 93%). ^77^

Our study had several limitations, as it did not account for the possibility of subjects having another infection. In this scenario, where all subjects are identified with a bacterial infection, it does not rule out the possibility of one having an additional infection. An acute infection that might have been caused by a viral agent could lead to alterations in the levels of IL-6 and IL-10. The increased IL-6 level may be followed by a decrease in IL-10, as it acts as a regulator. This could result in some subjects presenting with higher IL-6 and lower IL-10 levels than others.

## Conclusions

Lactoferrin in ONS intervention improved immune response homeostasis by balancing IL-6 and IL-10 and improved the IL-6/IL-10 ratio, not only body weight but also body length.

## Consent

Written informed consent for publication of the patients’ details was obtained from the parents of the patients.

## Data availability

### Underlying data

Figshare: Underlying data for ‘Effect of Lactoferrin in Oral Nutrition Supplement (ONS) towards IL-6 and IL-10 in Failure to Thrive Children with Infection’, https://www.doi.org/10.6084/m9.figshare.21813975.v2. ^44^

This project contains the following underlying data:

• Data file: Table 1: Data for Manuscript Effect of Lactoferrin in Oral Nutrition Supplement (ONS) towards IL-6 and IL-10 in Failure to Thrive Childre.xlsx
• Data archive 1: Elisa IL-6 Pre Intervention.rar
  ○ The concentration of IL-6 ng per L, pre intervention.pdf
  ○ Result of OD + Code.pdf
  ○ Result of OD Excel.xls
  ○ Result of OD.pdf
  ○ Standard curve.pdf
  ○ Sample scheme & Standard.pdf
• Data archive 2: Elisa IL-6 Post Intervention.rar
  ○ The concentration of IL-6, ng per L.pdf
  ○ Result of OD + Code.pdf
  ○ Result of OD Excel.xls
  ○ Result of OD.pdf
  ○ Standard curve.pdf
  ○ Sample scheme & Standard.pdf
• Data archive 3: Elisa_IL-10 Pre Intervention.rar
  ○ Result of concentration IL-10, pg per ml.pdf
  ○ Result of OD + Code.pdf
  ○ Result of OD Excel.xls
  ○ Result of OD.pdf
  ○ Standard curve.pdf
  ○ Sample scheme & Standard.pdf
• Data archive 4: Elisa_IL-10 Post Intervention.rar
  ○ Result of calculation concentration pg per ml.pdf
  ○ Result of OD + Code.pdf
  ○ Result of OD Excel.xls
  ○ Result of OD.pdf
  ○ Standard curve.pdf
  ○ Sample scheme & Standard.pdf

Data are available under the terms of the Creative Commons Zero “No rights reserved” data waiver (CC0 1.0 Public domain dedication).

### Extended data

Figshare: Extended data for ‘Effect of Lactoferrin in Oral Nutrition Supplement (ONS) towards IL-6 and IL-10 in Failure to Thrive Children with Infection’, https://www.doi.org/10.6084/m9.figshare.22210798.v3. ^45^

This project contains the following extended data:

• Informed consent: Essential information for potential research participants (WHO-CIOMS 2016)
• Airlangga University: Ethical clearance
• ClinicalTrials.gov: Protocol registration
• ClinicalTrials.gov: Completed study
• Study protocol

### Reporting guidelines

Figshare: TREND checklist for ‘Effect of Lactoferrin in Oral Nutrition Supplement (ONS) towards IL-6 and IL-10 in Failure to Thrive Children with Infection’, https://www.doi.org/10.6084/m9.figshare.22210798.v3. ^45^

Data are available under the terms of the Creative Commons Attribution 4.0 International license (CC-BY 4.0)
