# Supplementary material for: Effect of lactoferrin in oral nutrition supplement (ONS) towards IL-6 and IL-10 in failure to thrive children with infection
Source: F1000Res. 2023 Dec 19;12:897. Originally published 2023 Jul 28. [Version 3] doi: 10.12688/f1000research.130176.3 (PMC10904948; doi:10.12688/f1000research.130176.3)
Supplement: Peer review history

# Reviewer report: Reviewer response for version 3

**DOI:** 10.5256/f1000research.160100.r231437

**Published:** 2024-02-08

**Authors:** Rachael Anyim, Mallory Peters

**Affiliations:** 1 Anthropology, Binghamton University, Binghamton, New York, USA

**General Response:**

 We would like to thank the authors for their immense efforts to address our previous comments. We are thoroughly impressed with the improvements in flow and clarity. The concerns do not impact comprehension of the article’s core results. However, we highly recommend the authors incorporate these suggestions into their manuscript.

**Major Comments:**

1. A major component that is missing from the methods was disease burden tracking/monitoring of the course of disease progression for the sample participants. If information on the resolution of viral infections was not collected, its absence should be included in the limitations section. As stated, all participants were identified as “[failing] to thrive due to infectious illness” but it was never mentioned whether any of the participants' infections were resolved over the course of the intervention. Since disease burden is the identifying variable for this population, this is critical information, especially since individuals whose infections cleared over the period of observation would likely make significantly higher gains in weight and length/height. It is important to not conflate normal growth gains due to aging/growth and development and reduced disease burden with the impact of the ONS.

**Minor Comments:**

- Statements regarding Nigerian, Gazan, and Indonesian children in the intro (paragraph 2) is still out of place, as it’s discussing results. We recommend that the authors remove the following clauses and rephrase accordingly for clarity, as they have not discussed methods and should not be discussing generated results at this point:  "....which was higher compared to this study" ; "the prevalence of stunted/severely stunted children was higher in group-1 compared to group-2 in our study..."
- The following clause should not stand alone as a sentence and should be rephrased: “While the prevalence of underweight in two- to five-year-old children population in Gaza was 19.6%.”
- Intro paragraph 4 - typo - “,which supports by another finding” . The authors can either remove “by” or rephrase slightly to say “which is supported by” (or something similar in nature)
- Unclear where this study is occurring until intro paragraph 5 
- Unclear statement in methods, before “interventions” - “The sample size was 80 subjects; pre- and post-design needed for the study was 80 samples.” Does this mean there were 160 total samples? A pre- and post-intervention sample from each participant? Is this including participant withdrawals?
- Typo in methods, before “results” - “We hypotheses that” - should be “we hypothesize that”

Is the work clearly and accurately presented and does it cite the current literature?

Partly

If applicable, is the statistical analysis and its interpretation appropriate?

No

Are all the source data underlying the results available to ensure full reproducibility?

Yes

Is the study design appropriate and is the work technically sound?

Yes

Are the conclusions drawn adequately supported by the results?

Partly

Are sufficient details of methods and analysis provided to allow replication by others?

Partly

Reviewer Expertise:

Our combined areas of expertise involve human milk, maternal-infant health, biomarkers of stress (e.g., cortisol) and immunity/immune responses (e.g., cytokines, antibodies), anthropometry, human skeletal growth and plasticity, and biological specimen collection (including blood draws, saliva and milk collections), as well as enzyme immunoassay estimations.

We confirm that we have read this submission and believe that we have an appropriate level of expertise to confirm that it is of an acceptable scientific standard.

# Reviewer report: Reviewer response for version 3

**DOI:** 10.5256/f1000research.160100.r231436

**Published:** 2024-01-02

**Authors:** Vinutha U. Muktamath

**Affiliations:** 1 University of Agricultural Sciences, Dharwad, India

The article can be accepted.

Is the work clearly and accurately presented and does it cite the current literature?

Yes

If applicable, is the statistical analysis and its interpretation appropriate?

Partly

Are all the source data underlying the results available to ensure full reproducibility?

Yes

Is the study design appropriate and is the work technically sound?

Yes

Are the conclusions drawn adequately supported by the results?

Partly

Are sufficient details of methods and analysis provided to allow replication by others?

Yes

Reviewer Expertise:

Assessment of children, Differently abled and elderly

I confirm that I have read this submission and believe that I have an appropriate level of expertise to confirm that it is of an acceptable scientific standard.

# Reviewer report: Reviewer response for version 2

**DOI:** 10.5256/f1000research.155554.r217435

**Published:** 2023-11-06

**Authors:** Rachael Anyim, Mallory Peters

**Affiliations:** 1 Anthropology, Binghamton University, Binghamton, New York, USA

Many thanks to the authors for taking time to review and incorporate our suggestions into their manuscript. We see that they have taken our feedback under consideration, which is greatly appreciated. However, we noticed that the revised manuscript was not executed in a way that improved clarification. For example, discussion of the prevalence in different countries was moved to the introduction without actually revising the content, thereby presenting results before methods, which is inappropriate. We have provided additional comments towards targeted improvements. Please see below for “Major Comments” and “Minor Comments”.

**Major Comments:**

1. The entire manuscript needs grammatical revisions, including sentence restructuring. There are a number of errors that need to be addressed as their frequency and severity are hindering reader comprehension. Note, this is not an exhaustive list of examples: “While the prevalence in two- to five-year-old children in Gaza was 19.6%.11”. “Interleukin-10 (IL-10) is an anti-inflammatory cytokines with an essential roles to damped and minimalizing the damage due to pro-inflammatory response21 by inhibiting the activation of lymphocyte T to end up the immune reaction.” “The prevalent of tuberculosis (TB) is high in Indonesia, even increase during…” “We hypotheses that” 
2. As previously mentioned, hypothesis driven research should begin with clear hypotheses presented in the introduction. While the authors have added hypotheses, they have not been contextualized with appropriate background literature to support the proposed relationships. As currently presented, the hypotheses are actually results that have been rephrased as hypothetical statements which is not appropriate project formulation. If the authors did not already have hypotheses formulated prior to data collection and analyses, then the analyses needs to be restructured for an exploratory protocol and therefore rerun with appropriate tests for such a study [e.g., model selection/information theoretic approach using akaike information criterion corrected (AICc) or bayesian information criterion (BIC)].
3. A major component that is missing from the methods was disease burden tracking for the sample participants. As stated, all participants were identified as “[failing] to thrive due to infectious illness” but it was never mentioned whether any of the participants' infections were resolved over the course of the intervention. Since disease burden is the identifying variable for this population, this is critical information, especially since individuals whose infections cleared over the period of observation would likely make significantly higher gains in weight and length/height. It is important to not conflate normal growth gains due to aging/growth and development and reduced disease burden with the impact of the ONS.
4. There needs to be a “Limitations” section to address some of the pitfalls of the study. For example, the authors could use this section to discuss the issue of doing non-fasting blood draws in the evening when the biomarkers of interest may be subject to dietary fluctuations or to diurnal variation. While this was addressed in the response to reviewers, it should be included in the text of the manuscript, so that readers are given a complete picture of the methods and the associated factors that may confound results. 
5. As mentioned in our first review of this manuscript, the results presented in the abstract and results/discussion sections of the manuscript seem to be conflicting. Without having a control (or a healthy population, or other predetermined baselines attributing to what should be expected), there is no way to determine whether the lactoferrin and ONS is responsible for “improving” the weight gains or immune responses.
6. The authors directly addressed many of our previous comments from the original version of the manuscript in their response to the reviewer report. However, they neglected to revise those comments in the updated resubmission. For example: “Main Complaints” is still not addressed in the methods, so it is unclear how these complaints were documented/assessed (e.g., via survey at the beginning of study) and whether these complaints occurred during the course of the trial. Your response to us in the report “The complaints why the subjects visited the doctor were asked in the beginning by the nurse, “such as: what’s wrong with the baby, ma’am”” should be clarified in the methods section.

**Minor Comments:**

1. Introduction paragraph 4 concludes by stating that infection correlates with malnutrition degree but uses WAZ or HAZ as a metric of malnutrition which is not an appropriate analogous metric (growth impairment is not a direct measure of malnourishment, rather, it is a symptom of it). Instead, risk of infection correlates with degree of growth impairment based on WAZ or HAZ.
2. Improper citations: Paragraph 5 introduction - “Urinary tract infection (UTI) contributed to the incidence of stunting in children, as it causes anorexia which leads to a stagnant or inadequate weight gain.28” in which reference 28 is “Mckenna L, Sari AH, Mane S, et al.: Pediatric Tuberculosis Research and Development: Progress. Priorities and Funding Opportunities. Pathogens. 2022; 11: 128” makes no mention of UTI’s or anorexia and therefore this is an unsupported and potentially incorrect statement which cannot be corroborated.
3. Typographical errors/misspellings: “kwarshiorkhor” should be “kwashiorkor”
4. Move Table 7 (normality and homogeneity) to table 2, immediately after sample breakdown
5. The headings for columns 4 in table 5 and table 6 seem to be incorrectly labeled. We previously flagged table 6 as needing to have a ΔIL6/IL10 ratio. Instead, it would appear that the authors have mistakenly labeled the Δ in table 5, which does not seem accurate.

Is the work clearly and accurately presented and does it cite the current literature?

Partly

If applicable, is the statistical analysis and its interpretation appropriate?

No

Are all the source data underlying the results available to ensure full reproducibility?

Yes

Is the study design appropriate and is the work technically sound?

Yes

Are the conclusions drawn adequately supported by the results?

Partly

Are sufficient details of methods and analysis provided to allow replication by others?

Partly

Reviewer Expertise:

Our combined areas of expertise involve human milk, maternal-infant health, biomarkers of stress (e.g., cortisol) and immunity/immune responses (e.g., cytokines, antibodies), anthropometry, human skeletal growth and plasticity, and biological specimen collection (including blood draws, saliva and milk collections), as well as enzyme immunoassay estimations.

We confirm that we have read this submission and believe that we have an appropriate level of expertise to confirm that it is of an acceptable scientific standard, however we have significant reservations, as outlined above.

# Reviewer report: Reviewer response for version 2

**DOI:** 10.5256/f1000research.155554.r217434

**Published:** 2023-10-30

**Authors:** Vinutha U. Muktamath

**Affiliations:** 1 University of Agricultural Sciences, Dharwad, India

The article has been revised and the discussion is improvised. But again the mention in the sample size says the sample size was 80 subjects; pre- and post-design needed for the study was 160 samples. The 160 gives a meaning that there were 160 different samples. But the same 80 will be for pre and post-test. So mention the sample size was 80 and they were tested at baseline(pretest) and Post-test after intervention.

 The hypothesis should be stated as either a null hypothesis or an alternative hypothesis. There should be 3-4 hypotheses and very simple

 Example

**Null hypothesis**-Lactoferrin in ONS does not improve body weight and length,

 Lf supplementation in ONS does not improve immune response homeostasis by balancing IL-6 and IL-10 levels and by improving the IL-6/IL-10 ratio.

**Alternative Hypothesis**

 -Lactoferrin in ONS  improves body weight and length,

 Lf supplementation in ONS improves immune response homeostasis by balancing IL-6 and IL-10 levels and by improving the IL-6/IL-10 ratio.

Is the work clearly and accurately presented and does it cite the current literature?

Yes

If applicable, is the statistical analysis and its interpretation appropriate?

Partly

Are all the source data underlying the results available to ensure full reproducibility?

Yes

Is the study design appropriate and is the work technically sound?

Yes

Are the conclusions drawn adequately supported by the results?

Partly

Are sufficient details of methods and analysis provided to allow replication by others?

Yes

Reviewer Expertise:

Assessment of children, Differently abled and elderly

I confirm that I have read this submission and believe that I have an appropriate level of expertise to confirm that it is of an acceptable scientific standard, however I have significant reservations, as outlined above.

# Response

**Published:** 2023-11-01

**Authors:** Nur Aisiyah Widjaja

**Affiliations:** Airlangga University, Indonesia

**Reviewer notes 1:**

 The article has been revised and the discussion is improvised. But again the mention in the sample size says the sample size was 80 subjects; pre- and post-design needed for the study was 160 samples. The 160 gives a meaning that there were 160 different samples. But the same 80 will be for pre and post-test. So mention the sample size was 80 and they were tested at baseline(pretest) and Post-test after intervention.

**Response:**

 Dear Sir,

 We are sorry for not understanding the suggestion in the first review. Here we made the changes in the manuscript.

**Reviewer notes 2:**

 The hypothesis should be stated as either a null hypothesis or an alternative hypothesis. There should be 3-4 hypotheses and very simple

 Example

 Null hypothesis-Lactoferrin in ONS does not improve body weight and length,

 Lf supplementation in ONS does not improve immune response homeostasis by balancing IL-6 and IL-10 levels and by improving the IL-6/IL-10 ratio.

 Alternative Hypothesis

 -Lactoferrin in ONS improves body weight and length,

 Lf supplementation in ONS improves immune response homeostasis by balancing IL-6 and IL-10 levels and by improving the IL-6/IL-10 ratio.

**Response:**

 Dear Sir. Thank you for your suggestion to shorten our hypothesis. Here we choose to use the second suggestion (H alternatives).

1. Lactoferrin in ONS improves body weight and length/height.
2. Lactoferrin supplementation in ONS improves immune response homeostasis by balancing IL-6 and IL-10 levels, and by improving the IL-6/IL-10 ratio.
3. At least there was a pair of groups of nutritional status had a significant difference of IL-6 and IL-10 levels, and the IL-6/IL-10 ratio.

# Reviewer report: Reviewer response for version 1

**DOI:** 10.5256/f1000research.142910.r197396

**Published:** 2023-08-30

**Authors:** Rachael Anyim, Mallory Peters

**Affiliations:** 1 Anthropology, Binghamton University, Binghamton, New York, USA

Widjaja et al.’s manuscript highlights growth failure in children (diagnosed with infectious diseases) and whether lactoferrin in oral nutrition supplementation improves their immune responses, captured here via changes in pro-inflammatory and anti-inflammatory cytokines, interleukin-6 and interleukin-10, respectively. Participating children received lactoferrin in oral nutrition supplementation for a period of 90 days, which the authors claim resulted in increased body length and weight, as well as decreased interleukin-10 and interleukin-6 (though the latter was not statistically significant between pre- and post-intervention). Furthermore, children with severe growth stunting experienced a larger decrease in interleukin-6 compared to children of normal stature.

 As a whole, the authors’ manuscript is very detailed and well written, evidenced by their thorough references to the current literature. The methods for data collection are written clearly for replication and the study is well executed. However, the paper could benefit from some reorganizing. For example, “stunting” is defined on page 12 in the middle of paragraph 3 but should be discussed much earlier in the paper (i.e., in the introduction). The statistics presented also need a significant overhaul. See “Major Comments” and “Minor Comments” for additional details.  

**Major Comments:**

1. The introduction section should be expanded to include functions/relevance of interleukin-10 (not just IL-6 and lactoferrin), definitions of stunting versus wasting, and outline the growth standards used. It could also benefit from highlighting the study scope and setting. For example, in the first paragraph in the discussion section (page 11), it is unclear why the authors discuss the prevalence of stunting in other countries when these have not been previously addressed. It might be better to move this information to the introduction to improve the background information presented and flesh out the current relevance to Indonesia in particular. It could be useful to state the rationale for conducting this study in Indonesia, what infectious disease burdens are prevalent in the area (e.g., urinary tract infections and tuberculosis are mentioned in the methods but then COVID-19 and pneumonia are in the discussion) and whether these may be confounding variables in analyses. If available within the data they collected, the authors could also address cultural considerations since part of the intervention includes dietary counseling and dietary supplementation of animal protein, as well as whether participants are still breastfeeding and how this might impact the generated results.
2. While the authors have cited the literature, they have not stated what hypotheses may be driving their analyses and what results they expect to yield. These should be foregrounded in the introduction for readers.
3. Methods for data collection, including anthropometrics and blood draws, seem appropriate. However, it would have been good to see a discussion on the volume of blood drawn and whether it was a fasting draw, as well as information on time of day for collections since some bioactive properties (e.g., IL-6) can exhibit diurnal variation, thereby confounding the results. The authors have only one sentence detailing their statistical analyses and superscripts in corresponding tables, which severely limits the replicability of their quantitative methods. It would be useful to expand on their methods here - including the results of normality and homogeneity tests and clearly describing which tests were performed on which variables, so that there is no confusion as to how analyses were conducted. 
4. Clarification on the results presented would be helpful; as currently written, there seem to be mismatches between the statistics and the authors’ reports/concluding statements. We wonder if, perhaps, different tests are reported in the text versus in the tables; if so, the results yielded in those tests should match those in the tables/text. For example, in the abstract the authors suggest that IL-10 was significantly reduced after intervention. However, the closest value to statistical significance is p-value = 0.076 presented in Table 5. Additionally, they state that their intervention improved the IL-6/IL-10 ratio. However, there are no statistically significant differences in pre- and post-intervention ratios, as seen in Table 6. Convention is moving beyond solely using p < 0.05 as a threshold for data interpretation, so if that is the case here, the authors should disclose their cutoff point for determining “significant” results. Furthermore, the authors should remodel their tables to not only include p-values but also to include the comprehensive test results, test statistics, and effect sizes/magnitude of the effect. The p-values, means, and standard deviations presented do not convey a complete picture of the story written in the abstract and in the paper itself. Additionally, presenting SD as a +/- value suggests symmetry of data without actually testing for it.  It could be useful if the authors instead did repeated measures ANOVA of pre- and post-intervention within group changes for IL-6 and IL-10, as well as present the tests of normality and homogeneity and the results of those tests. Additionally, clarification on the specific growth curves being used and how it was determined that Oral Nutrition Supplement intervention improved body weight and length/height could also be useful. For example, Table 1/paragraph 1 states that age, body weight, and height age were lower in group 1 versus group 2. However, the groups were broken up by age, so these changes could largely be innate and not necessarily linked to ONS supplementation. Table 2 presents a similar issue. Were the authors comparing these metrics to external growth standards? If so, those standards are not clearly identified here (aside from mentioning WHO Anthro). If not, how were the authors able to confirm whether the growth is an actual improvement of health due to the ONS intervention and not the result of normal growth over the three-month period? Perhaps, it may also be useful to add the rationale in age group cutoffs (i.e., group 1 vs. group 2).

**Minor Comments:**

1. Sentence Structure: In the introduction section’s first paragraph (page 3), the authors can remove “still”, as it presupposes the audience is aware of growth failure as a historic health problem. In paragraph two, the second to last sentence serves no clear purpose and should either be expanded or removed. The first 2 sentences in the third paragraph can be consolidated a bit. The first sentence in the last paragraph on page 11 (“Lactoferrin…act accordingly and limits tissue damage”) doesn’t seem to make sense with the last clause. This may be due to missing words or punctuation.
2. Typographical Errors and Points of Clarification: In the discussion section, “secrets” should be “secretes” in the twelfth paragraph (page 13). “Main Complains”, in Table 1, should be “Main Complaints”. Furthermore, “Main Complaints” were not addressed in the methods, so it is unclear how these complaints were documented/assessed (e.g., via survey at the beginning of study) and whether these complaints occurred during the course of the trial. “Liquid pups” should also be corrected in the table. Weight faltering (“normo-weight”) is unclearly defined in the table and in the text of the paper. In the far right column of Table 6, the column is labeled as ΔIL-10 but should be ΔIL-6/IL-10. It could also be useful if the authors clarified in the methods how animal protein supplementation was conducted, the rationale behind it, and how its application was measured.

Overall, the study design and work presented have academic merit, as well as public health implications. If, for example, a relatively inexpensive intervention demonstrates positive health outcomes, this is a valid justification for using it in potentially low-income, nutrient impoverished, or high disease-burden settings. However, for the reasons stated above, it is a little difficult to accurately assess whether there is a reduction in IL-6 and if this is indeed directly related to the intervention.

Is the work clearly and accurately presented and does it cite the current literature?

Partly

If applicable, is the statistical analysis and its interpretation appropriate?

No

Are all the source data underlying the results available to ensure full reproducibility?

Yes

Is the study design appropriate and is the work technically sound?

Yes

Are the conclusions drawn adequately supported by the results?

Partly

Are sufficient details of methods and analysis provided to allow replication by others?

Partly

Reviewer Expertise:

Our combined areas of expertise involve human milk, maternal-infant health, biomarkers of stress (e.g., cortisol) and immunity/immune responses (e.g., cytokines, antibodies), anthropometry, human skeletal growth and plasticity, and biological specimen collection (including blood draws, saliva and milk collections), as well as enzyme immunoassay estimations.

We confirm that we have read this submission and believe that we have an appropriate level of expertise to confirm that it is of an acceptable scientific standard, however we have significant reservations, as outlined above.

# Response

**Published:** 2023-09-02

**Authors:** Nur Aisiyah Widjaja

**Affiliations:** Airlangga University, Indonesia

As a whole, the authors’ manuscript is very detailed and well written, evidenced by their thorough references to the current literature. The methods for data collection are written clearly for replication and the study is well executed. However, the paper could benefit from some reorganizing. For example, “stunting” is defined on page 12 in the middle of paragraph 3 but should be discussed much earlier in the paper (i.e., in the introduction). The statistics presented also need a significant overhaul. See “Major Comments” and “Minor Comments” for additional details. 

**Response: **Paragraph 2 sentences 2, and soon

 Major Comments:

     The introduction section should be expanded to include functions/relevance of interleukin-10 (not just IL-6 and lactoferrin), definitions of stunting versus wasting, and outline the growth standards used.

**Response: **Noted: Paragraph 2 sentences 2 to 3.

 It could also benefit from highlighting the study scope and setting. For example, in the first paragraph in the discussion section (page 11), it is unclear why the authors discuss the prevalence of stunting in other countries when these have not been previously addressed. It might be better to move this information to the introduction to improve the background information presented and flesh out the current relevance to Indonesia in particular.

**Response: **Noted: Paragraph 2 sentences 4 until the last paragraph. 

 It could be useful to state the rationale for conducting this study in Indonesia, what infectious disease burdens are prevalent in the area (e.g., urinary tract infections and tuberculosis are mentioned in the methods

**Response: ** Noted. Paragraph 5. 

 But then COVID-19 and pneumonia are in the discussion) and whether these may be confounding variables in analyses.

**Response: **We add COVID-19 due to during the intervention, we found that our subjects were infected. As for pneumonia, we found this infection in several subjects. We thought those disease as the confounding factors, but after we analysed statistically, they didn’t (p>0.05).

 If available within the data they collected, the authors could also address cultural considerations since part of the intervention includes dietary counseling and dietary supplementation of animal protein, as well as whether participants are still breastfeeding and how this might impact the generated results.

**Response: **All of the subjects had breastfed predominantly (those under 2 years old), but the older ones consume growing up formula.

 While the authors have cited the literature, they have not stated what hypotheses may be driving their analyses and what results they expect to yield. These should be foregrounded in the introduction for readers.

**Response: **Noted: The last sentences in introduction, and hypotheses in the methods.

 Methods for data collection, including anthropometrics and blood draws, seem appropriate. However, it would have been good to see a discussion on the volume of blood drawn and whether it was a fasting draw, as well as information on time of day for collections since some bioactive properties (e.g., IL-6) can exhibit diurnal variation, thereby confounding the results.

**Response: **Noted: Paragraph 2 sub heading intervention in Method. Due to the researchers working during evening until night, the subjects were taken the blood at that time by the laboratory employee accompanied by the doctor’s nurses without fasting.

 The authors have only one sentence detailing their statistical analyses and superscripts in corresponding tables, which severely limits the replicability of their quantitative methods. It would be useful to expand on their methods here - including the results of normality and homogeneity tests and clearly describing which tests were performed on which variables, so that there is no confusion as to how analyses were conducted.

**Response: ** Noted: Table 7

 Clarification on the results presented would be helpful; as currently written, there seem to be mismatches between the statistics and the authors’ reports/concluding statements. We wonder if, perhaps, different tests are reported in the text versus in the tables; if so, the results yielded in those tests should match those in the tables/text. For example, in the abstract the authors suggest that IL-10 was significantly reduced after intervention. However, the closest value to statistical significance is p-value = 0.076 presented in Table 5. Additionally, they state that their intervention improved the IL-6/IL-10 ratio. However, there are no statistically significant differences in pre- and post-intervention ratios, as seen in Table 6. Convention is moving beyond solely using p < 0.05 as a threshold for data interpretation, so if that is the case here, the authors should disclose their cutoff point for determining “significant” results. Furthermore, the authors should remodel their tables to not only include p-values but also to include the comprehensive test results, test statistics, and effect sizes/magnitude of the effect. The p-values, means, and standard deviations presented do not convey a complete picture of the story written in the abstract and in the paper itself. Additionally, presenting SD as a +/- value suggests symmetry of data without actually testing for it.  It could be useful if the authors instead did repeated measures ANOVA of pre- and post-intervention within group changes for IL-6 and IL-10, as well as present the tests of normality and homogeneity and the results of those tests. Additionally, clarification on the specific growth curves being used and how it was determined that Oral Nutrition Supplement intervention improved body weight and length/height could also be useful. For example, Table 1/paragraph 1 states that age, body weight, and height age were lower in group 1 versus group 2. However, the groups were broken up by age, so these changes could largely be innate and not necessarily linked to ONS supplementation. Table 2 presents a similar issue. Were the authors comparing these metrics to external growth standards? If so, those standards are not clearly identified here (aside from mentioning WHO Anthro). If not, how were the authors able to confirm whether the growth is an actual improvement of health due to the ONS intervention and not the result of normal growth over the three-month period? Perhaps, it may also be useful to add the rationale in age group cutoffs (i.e., group 1 vs. group 2).

**Response: **: Dear reviewer, the statement of IL-10 was significantly reduced after the intervention refers to a whole subject’s analysis using Wilcoxon sign rank (Komogorv-Smirnorv, p<0.05). We did not mention the value due to the limitations

 IL-6 before: 128.45 + 109.92

 IL-6 after: 111.76 + 78.10

 p=0.554

 IL-10 before: 461.20 + 392.12

 IL-10 after: 261.28 + 163.97

 p=0.000

 IL-6/IL-10 ratio before: 0.33 + 0.22

 IL-6/IL-10 ratio after: 0.44 + 0.11

 p=0.000

 as for stated in the table, it was the value after we divided them based on the hypotheses we had.

 Minor Comments:

 Sentence Structure: In the introduction section’s first paragraph (page 3), the authors can remove “still”, as it presupposes the audience is aware of growth failure as a historic health problem.

**Response: **Noted. We have erased it

 In paragraph two, the second to last sentence serves no clear purpose and should either be expanded or removed. The first 2 sentences in the third paragraph can be consolidated a bit. The first sentence in the last paragraph on page 11 (“Lactoferrin…act accordingly and limits tissue damage”) doesn’t seem to make sense with the last clause. This may be due to missing words or punctuation.

**Response: **Noted. 

 Typographical Errors and Points of Clarification: In the discussion section, “secrets” should be “secretes” in the twelfth paragraph (page 13). “Main Complains”, in Table 1, should be “Main Complaints”.

**Response: **Noted.

 Furthermore, “Main Complaints” were not addressed in the methods, so it is unclear how these complaints were documented/assessed (e.g., via survey at the beginning of study) and whether these complaints occurred during the course of the trial.

**Response: **: The complaints why the subjects visited the doctor were asked in the beginning by the nurse, “such as: what’s wrong with the baby, ma’am”

 “Liquid pups” should also be corrected in the table. Weight faltering (“normo-weight”) is unclearly defined in the table and in the text of the paper. In the far-right column of Table 6, the column is labeled as ΔIL-10 but should be ΔIL-6/IL-10.

**Response: **Noted. We termed it as weight faltering because WAZ categories in normal range based on WHO child growth standard, but the subjects experienced inadequate weight gain

 It could also be useful if the authors clarified in the methods how animal protein supplementation was conducted, the rationale behind it, and how its application was measured.

**Response: **ONS Oral Nutrition Supplement / High Calorie Formula / Nutrient Densed Formula SGM Optic Grow contains animal protein with the protein energy ratio of 9.5% (according to WHO, to increase weight gain for catch-up growth 8.5% - 11.5% is needed).

 The measurement was made based on the administration compliance of high-density formula (ONS)

 Aside from ONS, subject is also given nutritional counseling to give one egg per day as an additional complementary feeding.

# Reviewer report: Reviewer response for version 1

**DOI:** 10.5256/f1000research.142910.r197395

**Published:** 2023-08-29

**Authors:** Vinutha U. Muktamath

**Affiliations:** 1 University of Agricultural Sciences, Dharwad, India

The study is novel and addresses the most important issue of poor nutritional status leading to infant mortality and morbidity in infants and toddlers. Appropriate statistical tool and methodology is adopted. However the title can be more specific as effect of lactoferrin on growth and stunting in children. And the sample size will be 80 and 160 as the same sample will be used for pre and post test.

 The interpretation and  discussion part needs to be rewritten as the present study is measuring only IL-6 and IL-10 levels in failure to thrive children. No where the results indicate the effect on immune system like reduction in morbidity status and increase in weight and length by morbidity status. More reviews can be added to support the effect of lactoferrin in oral nutrition on growth of children. By discussing the studies by other researchers on improvement of immune system cannot be used to conclude as" Lactoferrin in ONS intervention improved immune response homeostasis by balancing IL-6 and IL-10". Better to restrict to the results of present study

Is the work clearly and accurately presented and does it cite the current literature?

Yes

If applicable, is the statistical analysis and its interpretation appropriate?

Partly

Are all the source data underlying the results available to ensure full reproducibility?

Yes

Is the study design appropriate and is the work technically sound?

Yes

Are the conclusions drawn adequately supported by the results?

Partly

Are sufficient details of methods and analysis provided to allow replication by others?

Yes

Reviewer Expertise:

Assessment of children, Differently abled and elderly

I confirm that I have read this submission and believe that I have an appropriate level of expertise to confirm that it is of an acceptable scientific standard, however I have significant reservations, as outlined above.

# Response

**Published:** 2023-09-05

**Authors:** Nur Aisiyah Widjaja

**Affiliations:** Airlangga University, Indonesia

The study is novel and addresses the most important issue of poor nutritional status leading to infant mortality and morbidity in infants and toddlers. Appropriate statistical tool and methodology is adopted. However the title can be more specific as effect of lactoferrin on growth and stunting in children.

**Dear Mr. Vinutha U. Muktamathk, the reviewer of F1000Research**

**thank you for the suggestion for this article. Here the title we think of “Effect of lactoferrin in oral nutrition supplement (ONS) towards IL-6 and IL-10 on growth and stunting in children”**

 And the sample size will be 80 and 160 as the same sample will be used for pre and post-test.

**Thank you for the correction. We will change the sample size as you suggest**

 The interpretation and discussion part needs to be rewritten as the present study is measuring only IL-6 and IL-10 levels in failure to thrive children. No where the results indicate the effect on immune system like reduction in morbidity status and increase in weight and length by morbidity status. More reviews can be added to support the effect of lactoferrin in oral nutrition on growth of children. By discussing the studies by other researchers on improvement of immune system cannot be used to conclude as" Lactoferrin in ONS intervention improved immune response homeostasis by balancing IL-6 and IL-10". Better to restrict to the results of present study

**For growth parameters, we made other paper entitled “The Effect of High Calorie Formula on Weight, Height Increment, IGF-1 and TLC in Growth Faltering Children”. in this paper we add total lymphocyte count (TLC) as the immunological parameters in these children.**

**We will include the suggestion in the third version.**
